# A Combination of Biomarkers Predict Response to Immune Checkpoint Blockade Therapy in Non-Small Cell Lung Cancer

**DOI:** 10.3389/fimmu.2021.813331

**Published:** 2021-12-23

**Authors:** Zedong Jiang, Yao Zhou, Juan Huang

**Affiliations:** ^1^ Department of Hematology, Sichuan Academy of Medical Sciences & Sichuan Provincial People’s Hospital, University of Electronic Science and Technology of China, Chengdu, China; ^2^ College of Bioinformatics Science and Technology, Harbin Medical University, Harbin, China

**Keywords:** immune checkpoint blockade, immunotherapy, biomarker, biomarker combinations, non-small-cell lung cancer

## Abstract

Immune checkpoint blockade (ICB) therapy has provided clinical benefits for patients with advanced non-small-cell lung cancer (NSCLC), but the majority still do not respond. Although a few biomarkers of ICB treatment response have been developed, the predictive power of these biomarkers showed substantial variation across datasets. Therefore, predicting response to ICB therapy remains a challenge. Here, we provided a concise combinatorial strategy for predicting ICB therapy response and constructed the ICB treatment signature (ITS) in lung cancer. The prediction performance of ITS has been validated in an independent ICB treatment cohort of NSCLC, where patients with higher ITS score were significantly associated with longer progression-free survival and better response. And ITS score was more powerful than traditional biomarkers, such as TMB and PD-L1, in predicting the ICB treatment response in NSCLC. In addition, ITS scores still had predictive effects in other cancer data sets, showing strong scalability and robustness. Further research showed that a high ITS score represented comprehensive immune activation characteristics including activated immune cell infiltration, increased mutation load, and TCR diversity. In conclusion, our practice suggested that the combination of biomarkers will lead to a better prediction of ICB treatment prognosis, and the ITS score will provide NSCLC patients with better ICB treatment decisions.

## Introduction

Lung cancer is the most common malignant tumor in the world (1). In the current clinical practice, immune checkpoint therapy (2, 3) and combination therapy strategy (4, 5) has achieved amazing therapeutic effects in the treatment of cancer and have changed the clinical management of cancer. However, despite the strong improvement of these antibodies in cancer treatment, it is important to note that most patients fail to respond to ICBs or even have to stop treatment because of immune-related adverse events (6). Therefore, predicting ICB response is a key challenge in guiding patients to select current checkpoint immunotherapy and providing indicators of early treatment response.

Several biomarkers for predicting responsiveness to ICB treatment were developed. PD-L1 expression was one of the most promising biomarkers, which higher level of expression contributed better benefit. Nevertheless, some studies reported that advanced NSCLC patients with lower PD-L1 expression still benefit from anti-PD1 therapy (6–8). Tumor mutation burden (TMB) emerged as a biomarker for ICB patient stratification, in which abundant non-synonymous mutations lead to an increased number of neoantigen, potentially increasing immune recognition and response (9). Higher TMB is associated with improved prognosis and increased response rate to ICB therapies in most studies (9–11). In addition, several immune escape mechanisms hindered the application of ICB therapy (12). Decreased T-cell infiltration has been reported to be associated with a poorer prognosis (13), and TGF-β signal limits the infiltration of T cells, forming a suppressed immune microenvironment (14, 15). These mechanisms were also used to develop ICB response biomarkers, such as TIDE (16) and PAN-fibroblast TGF-β response signature (17).

Due to the complexity of factors affecting immune checkpoint therapy, current biomarkers mainly involve a single ICB response mechanism, which is not good in predicting the prognosis of non-small cell lung cancer immune checkpoint therapy (18). Therefore, it is still a meaningful topic to explore the prognostic markers of ICB in non-small cell lung cancer.

Therefore, in this study, we focused on building a combinatorial biomarker for ICB treatment in lung cancer. Firstly, we evaluated genes related to ICB response biomarkers including cytotoxic T lymphocyte (CTL) level, TMB, and TGF-β signal. Then, an ICB treatment score (ITS) for lung cancer was established by integrating these genes. The relationship of the ITS with immune activity and mutations was also analyzed. Finally, we found that the ITS is a powerful prognostic biomarker and predicts the response to immune checkpoint inhibitors.

## Method

### Data Acquisition

Gene expression with the format of fragments per kilobase million (FPKM) and mutation data of TCGA-LUAD and TCGA-LUSC sample were obtained from UCSC Xena (http://xena.ucsc.edu). For the NSCLC ICB treatment cohort, we collected gene expression and the corresponding clinical response data of ICB pre-treatment patients from the Jung et al. (19) study. We also validated the prognosis efficiency of ITS score in three additional datasets including Gide et al. (20), Van Allen et al. (21), and Mariathasan et al. (17), involving two other cancer types. RNA-seq data of these ICB treatment cohorts were obtained through the supplementary materials of original publications.

### Calculation of ICB Treatment Signature (ITS) Score

We used the average expression level of CD8A, CD8B, GZMA, GZMB, and PRF1 to characterize cytotoxic T lymphocyte (CTL) levels. The TGF-β signaling score was calculated based on signatures proposed by a previous study (22). By spearman’s rank correlation test, we screened genes associated with CTL and TGF-β signaling with cor greater than 0.3 and FDR less than 0.01. According to TMB, patients were equally divided into three groups, high, middle, and low, and the differential genes between TMB-high and TMB-low group screened by Wilcoxon rank-sum test were considered to be TMB-related genes. Pathway enrichment analysis of genes was carried out with a hypergeometric test described previously (23). We selected genes associated with both high CTL level and high TMB and excluded immunosuppression-related genes as the ICB treatment signature (ITS). For each sample, the ITS score was calculated by ssGSEA (24) with default parameters in the “GSVA” R package. The interaction network analysis of ITS genes was performed by the Metascape online tool (25).

### Robustness Evaluation of ITS

We assessed the robustness of the prognostic power of ITS using the following methods. Firstly, ITS scores calculated by GSVA (26), Zscore (27), and PLAGE (28) with default parameters were used to evaluate whether the prognostic efficacy of ITS depended on specific scoring methods. Then, we evaluated the prognostic efficacy of signatures derived from multiple combinations of genes related to CTL, TMB, and TGF-β to discuss the necessity of three factors. And we randomly selected genes from ITS 100 times in a fixed proportion, from 0.1 to 0.9, to discuss how much does the absence of some genes in ITS effects the predictive power.

### Immune-Related and Genomic Features of Lung Cancer Sample

We calculated and collected expression signatures, CIBERSORT fractions, DNA damage scores, intratumor heterogeneity (ITH), TCR/BCR diversity, stromal fraction, and Leukocyte fraction for each lung cancer sample (22). Expression signatures include proliferation, macrophages regulation, overall lymphocyte infiltration, TGF-β response, IFN-g response, and wound healing. The composition ratio of 22 immune cells in the sample was inferred using the CIBERSORT algorithm (29). Some immune cell types were aggregated to a major class including total lymphocytes, total dendritic cells, total macrophage, and total mast cells. These proportions were multiplied by leukocyte fraction, estimated from a mixture model with specific methylation probes, to yield corresponding estimates in terms of overall fraction in samples. ABSOLUTE algorithm (30) was used to calculate tumor purity, aneuploidy scores, and ITH. The stromal fraction was defined as the total non-tumor cellular component, obtained by subtracting tumor purity from unity.

### Screening Features Associated With ITS Scores

We used linear mixed-effects models to associate ITS score with TME features in lung cancers using the “lme4” R package. For each feature, we compared a model with the feature to a model without this feature using an ANOVA to determine whether the ITS score was significantly associated with this feature in lung cancers. We adjusted patient age and sex and set cancer type as a random effect in every model. This allowed us to consider a different baseline value for the feature in LUAD and LUSC. The conditional R2 was reported which reflects the variance explained by the fixed and random factors. An FDR adjustment was applied to the p-values from the linear mixed-effect model. Features with FDR less than 0.01 were considered to be correlated with ITS score.

### Statistics Analysis

All statistical analyses were performed with R statistical software version 3.5.2 (http://www.R-project.org). Wilcoxon rank-sum test was used to determine the significance of differences between the two groups. The correlation between the two continuous variables was evaluated by spearman’s rank correlation test. The survival curves were calculated with Kaplan-Meier estimation, and the differences between survival curves were calculated by log-rank test. The hazard ratio, multivariate analysis adjusting for clinical parameters was determined through a Cox proportional hazards model. Survival analysis was carried out using the “survminer” and “survival” R packages.

## Results

### Identification of Immune-Related Genes Used to Construct ICB Treatment Signature in Lung Cancer

**Figure 1:**
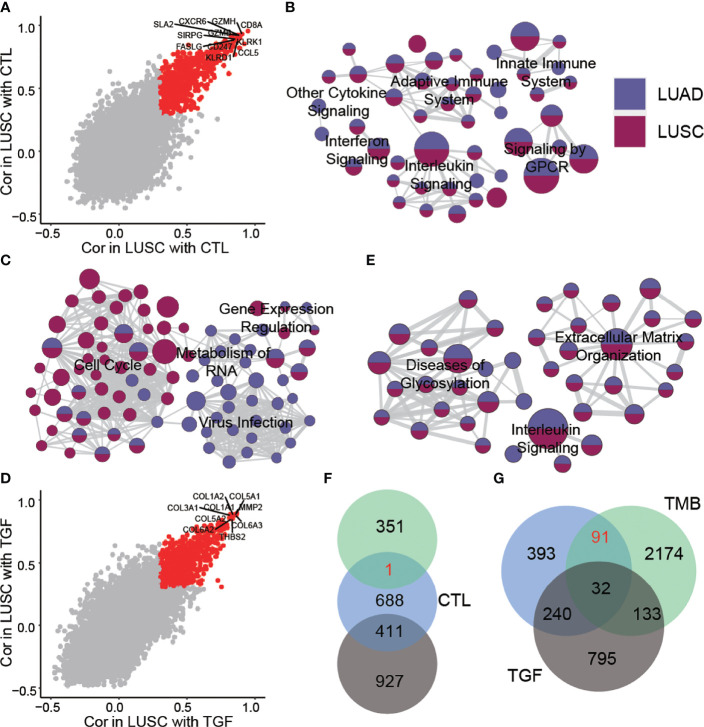
Genes and pathways related to ICB treatment response biomarker. **(A)** Genes related to CTL in LUAD and LUSC. The red dots represent an association greater than 0.3 in two cancer types. **(B)** Pathways related to CTL in LUAD and LUSC. **(C)** Pathways related to TMB in LUAD and LUSC. **(D)** Genes related to TGF-β in LUAD and LUSC. The red dots represent an association greater than 0.3 in two cancer types. **(E)** Pathways related to TGF-β in LUAD and LUSC. **(F)** Relationship between genes related to CTL, TMB, and TGF-β in LUSC. **(G)** Relationship between genes related to CTL, TMB, and TGF-β in LUAD.

To construct an integrated ICB response biomarker, we first evaluated genes and pathways associated with three mechanisms related to ICB response (CTL, TMB, and TGF-β signaling) in lung cancer samples (see Methods). We identified 756 and 1,100 genes associated with high CTL levels in lung adenocarcinoma (LUAD) and lung squamous carcinoma (LUSC), respectively, of which 608 genes were found in both cancers (**Figures 1A**, **S1A**). Pathway enrichment analysis showed that these genes converged to immune-related pathways, including innate immune system, adaptive immune system, and various cytokine pathways (**Table S1**, **Figure 1B**). The genes associated with TMB were significantly different between the two cancer types. A large number of genes in LUAD were associated with high levels of TMB compared to LUSC (**Figure S1B**). In LUAD, TMB-related genes were mainly enriched in RNA metabolism and regulatory pathways, while in LUSC, they were mainly enriched in the cell cycle pathway (**Table S1**, **Figure 1C**).

A large number of immunosuppressive-related genes were also identified in both cancer types (**Figures 1D**, **S1C**). These genes are associated with the abnormal glycosylation and extracellular matrix organization pathway (**Table S1**, **Figure 1E**). We found that the genes associated with high CTL infiltration and high TMB level still contained a certain number of immunosuppressive-related genes, which may have a poor contribution to the prognosis of immunotherapy, so we considered excluding these genes when constructing the biomarker. Finally, by combining three mechanisms related to ICB response, we selected genes associated with both high CTL level and high TMB and excluded immunosuppressive-related genes as the ICB treatment signature (ITS). In LUAD, ITS contained 91 genes (**Figure 1G**, **Table S2**), while in LUSC only one gene passed the screening criteria (**Figure 1F**), so we used ITS obtained from LUAD as a proxy for NSCLC. A close interaction network was formed between ITS genes, and we found three functional modules in the interaction network, which were respectively related to cell killing, interferon response, and transcription factor binding (**Figure S2**).

### ITS Score Predicts the Prognosis of Lung Cancer Patients With ICB Treatment

**Figure 2:**
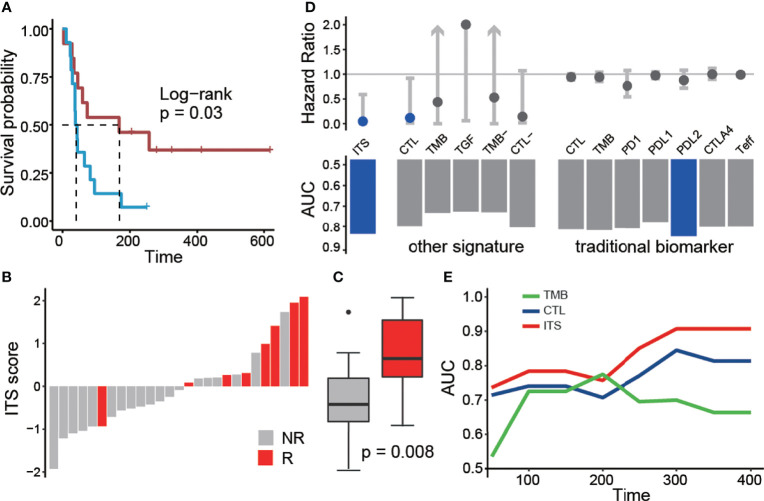
Predictive efficacy of ITS scores in an ICB treatment dataset for NSCLC. **(A)** PFS difference between the two groups with high and low ITS scores. **(B)** Response to treatment between the two groups with ITS score. Red indicates the patient’s response to treatment. **(C)** Differences in ITS scores between responders and non-responders. **(D)** Prognostic efficacy (top panel) and response prediction ability (bottom panel) of different signatures and biomarkers. **(E)** The area under the curve (AUC) was calculated for three prognostic models from 50 to 400 days. Blue: CTL; green: TMB; red: ITS.

Then, we used a cohort of NSCLC patients treated with ICB therapy to evaluate the prognosis efficacy of ITS. In this cohort, 85 genes in ITS had expression information. ITS score was estimated using single-sample gene set enrichment analysis (ssGSEA) with default parameters in each patient. Patients were divided into two groups based on median ITS scores. Patients with high ITS scores had significantly better progression-free survival (p = 0.03; **Figure 2A**) and higher objective response rates (53.8% *VS* 7.1%, p = 0.01; **Figure 2B**) than patients with low ITS scores. Meanwhile, the ITS score of responders was significantly higher than that of non-responders (p = 0.008; **Figure 2C**). Multivariate Cox analysis showed that the ITS score was an independent prognostic factor after adjustment for age and sex (HR = 0.097, p = 0.02). Alternatively, other algorithms such as PLAGE, GSVA, and Zscore could also be employed to calculate the ITS score. Patients with higher ITS scores could have better survival to immunotherapy (**Figure S3**). While for the PLAGE scores, higher scores may not indicate higher activities as PLAGE calculates the first principal component as the gene-set score.

To discuss whether the combination of CTL, TMB, and TGF-β is necessary to the predictive effectiveness in prognosis or response to immunotherapy, we built various signatures based on multiple combinations of these factors. Using these three factors alone, the only score of CTL-related genes had predictive power (**Figure 2D**). And the best predictive performance was achieved only when these factors were considered simultaneously (**Figure 2D**). We further evaluated the immunotherapy prognostic ability of each gene in ITS, and only 8.2% (7/85) genes had the prognostic ability (**Figure S4A**). we randomly selected genes from ITS 100 times in a fixed proportion to investigate the robustness of prognostic ability of ITS. It can be seen from the results that when 80% of the genes in ITS are lost, it can still ensure satisfactory prognostic efficacy (**Figure S4B**). It can be seen that these genes make ITS maintain very good stability in the application process because when we apply ITS, we cannot guarantee that every gene in ITS has expression information.

We also compared the prognosis efficacy of ITS with traditional biomarkers such as PD1, PD-L1, Teff, and TMB. None of these individual markers showed satisfactory predictive power, and only the ITS score was an independent prognostic factor for ICB treatment (**Table S3**, **Figure 2D**). Typically, CTL (p = 0.13; **Figure S5A**) and TMB (p = 0.36; **Figure S5B**) did not show significant prognosis efficacy of ICB treatment. And, the AUC of ITS was higher than that of CTL and TMB in predicting the survival rate of patients at different periods, showing a better prognosis efficiency (**Figure 2E**). In terms of predicting the response to ICB treatment, ICB also had a higher AUC than other biomarkers (**Table S3**). Taken together, the ITS score was demonstrated as a promising biomarker for immunotherapy after immune checkpoint inhibitors.

### ITS Score Represented Comprehensive Immune Characteristics

Further, we explored the biological explanation of the prognosis significance of the ITS score. The linear mixed-effect model was used to associate ITS score with TME and genomic features in lung cancers by adjusting for age and sex. LUAD samples were observed to have relatively high ITS scores than LUSC (**Figure S6A**). Therefore, cancer type was further incorporated as a random effect in the model, allowing us to consider a different baseline value for features in LUAD and LUSC.

Among the immune subtypes identified in previous studies (22), samples characterized by IFN-gamma dominance had the highest ITS scores, while lymphocyte-depleted samples possessed lower ITS scores (**Figure S6B**). Notably, C3 samples, which are predominantly immune-inflammatory and have the best prognosis, also had relatively low ITS scores, suggesting that some immune escape mechanism exists in these samples to influence the response to ICB treatment. We observed that proliferation rate (R^2^ = 0.269, FDR = 1.17e-41) and four immune expression signatures, macrophages (R^2^ = 0.565, FDR = 1.01e-80), lymphocyte infiltration (R^2^ = 0.615, FDR = 2.76e-113), IFN-g response (R^2^ = 0.462, FDR = 7.23e-61), and wound healing (R^2^ = 0.193, FDR = 2.49e-27), were more active in sample with higher ITS score (**Table S4**, **Figures S7**, **3A**).

**Figure 3:**
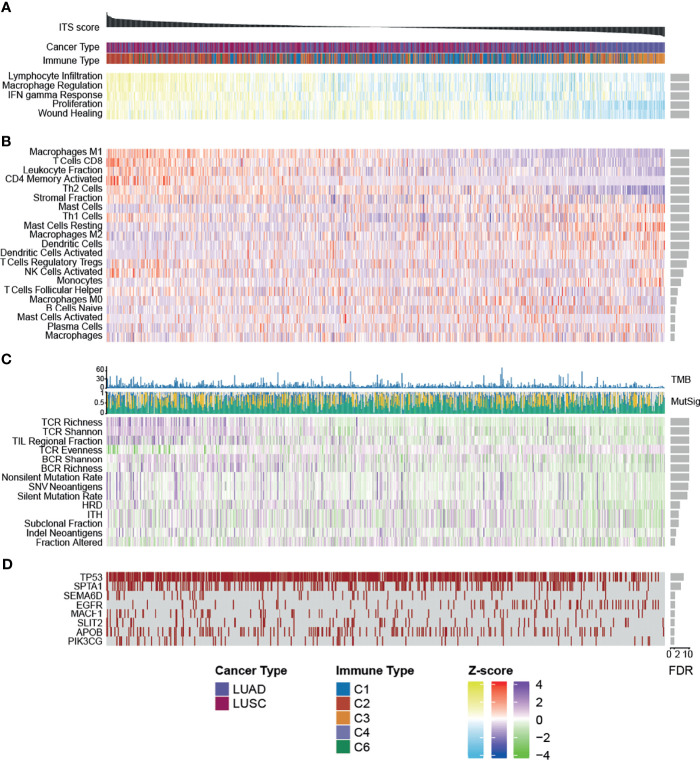
Heatmap showing the relationship between **(A)** expression signatures, **(B)** TME features, **(C)** tumor immunogenicity, and **(D)** mutations and ITS score across TCGA lung samples. Samples are ordered by ITS score.

We found that higher stromal fraction (R^2^ = 0.287, FDR = 9.06e-26) and leukocyte fraction (R^2^ = 0.355, FDR = 5.03e-60) were associated with stronger ITS score (**Table S4**, **Figure S7**, **Figure 3B**). Of the 29 classes of tumor-infiltrating immune cells tested, infiltrating levels of 21 showed significant related with ITS score (FDR < 0.01; **Table S4**). Of these, ten were highly infiltrated and eleven less enriched in tumors with elevated ITS score (**Figures S7**, **3B**). The sample with higher ITS score had abundant adaptive immune cells, including CD8+ T cells (R^2^ = 0.387, FDR = 7.01e-64), active Memory CD4 T cells (R^2^ = 0.370, FDR = 4.96e-55), and Th2 cells (R^2^ = 0.196, FDR = 4.27e-28). Low ITS score samples had more quiescent cells and activated innate immune cells, including resting mast cells (R^2^ = 0.219, FDR = 5.82e-16) and activated dendritic cells (R^2^ = 0.266, FDR = 2.10e-10), suggesting that patients in the low ITS group may have an insufficient adaptive immune response.

We also observed that samples with higher ITS score had increased tumor immunogenicity. For instance, elevated TCR richness (R^2^ = 0.459, FDR = 4.87-71), BCR richness (R^2^ = 0.199, FDR = 6.89e-19), TMB (R^2^ = 0.205, FDR = 1.24e-11), and SNV neoantigens (R^2^ = 0.199, FDR = 2.45e-10) were associated with high ITS score (**Figures S7**, **3C**). Considering the strong association of ITS score with mutational density, we next looked to determine whether these were selectively affected specific genes or chromosome regions. The mutation status of 69 genes was significantly correlated with the ITS score (FDR < 0.01; **Table S4**), of which 8 genes were related to immune or cancer genes (**Figure 3D**). Typically, mutations in the *EGFR* were associated with decreased ITS score (R^2^ = 0.156, FDR = 0.004), consistent with previous descriptions that *EGFR* mutant tumors have generally a low response to immune checkpoint inhibitors (31). And we detected no specific copy number region associated with the ITS score (**Table S4**), indicating a general effect of association.

Overall, these results suggested that the ITS score may represent comprehensive immune characteristics to predict the outcome of ICB treatment.

### Robustness of Prognosis Efficiency of ITS Score

**Figure 4:**
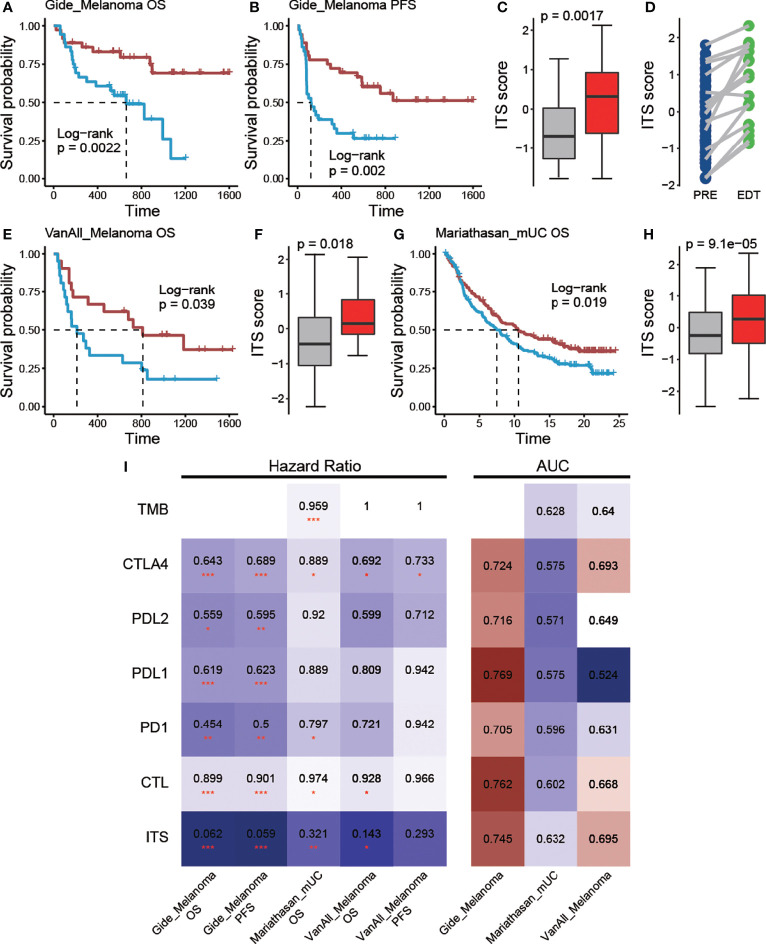
Predictive efficacy of ITS scores in other ICB treatment cohorts. **(A)** OS and **(B)** PFS difference between the two groups with high and low ITS scores in the Gide cohort. **(C)** Differences in ITS scores between responders and non-responders in the Gide cohort. **(D)** ITS score changes after treatment. **(E)** OS difference between the two groups with high and low ITS scores in the VanAll cohort. **(F)** Differences in ITS scores between responders and non-responders in the VanAll cohort. **(G)** OS difference between the two groups with high and low ITS scores in the Mariathasan cohort. **(H)** Differences in ITS scores between responders and non-responders in the Mariathasan cohort. **(I)** Biomarkers are shown as rows and individual cohorts as columns. The heatmap indicates the hazard ratio derived from Cox regression (left panel) and AUC value derived from ROC curve (right panel), * denoted p <0.05, ** denoted p <0.01, *** denotes p < 0.001.

To ensure the robustness of ITS scores for ICB treatment prognosis efficacy, it should be evaluated in more data sets. However, due to the lack of additional immunotherapy data for non-small cell lung cancer, we examined the prognostic efficacy of ITS scores for ICB treatment in other cancer types. In a melanoma cohort (20), immunotherapy-treated patients with higher ITS score demonstrated the longer OS (p = 0.002; **Figure 4A**) and PFS (p = 0.002; **Figure 4B**), and responders had significantly higher ITS scores than non-responders (p = 0.001; **Figure 4C**). In this dataset, some samples were also sequenced early during treatment (EDT), and we found that the ITS scores of almost all samples increased after treatment (**Figure 4D**), reflecting the dynamic changes of tumor immune microenvironment during ICB treatment. Similar findings were observed in another cohort of melanoma patients (21) (n = 42) treated with anti-CTLA-4 therapy. Patients with prolonged OS also noted in higher ITS score (p = 0.039; **Figure 4E**), and ITS score was significantly higher in responders and long-term survivors (p = 0.018; **Figure 4F**). In addition, ITS scores also showed prognostic efficacy for ICB treatment in bladder cancer (17). Immunotherapy-treated patients with higher ITS score also displayed a superior prognosis compared with the other patients (p = 0.019; **Figure 4G**), and respondents were associated with higher ITS scores (p = 9.1e-5; **Figure 4H**). In summary, these results indicated the robust and practical prognosis efficacy of the ITS score. We also compared the prognosis and response prediction efficacy of ITS with traditional biomarkers in these three cohorts. ITS still showed satisfactory results (**Figure 4I**), although it was not trained in the corresponding cancer sample. This further confirms the prognostic potential of the combined strategies.

## Discussion

In this study, we analyzed genes and pathways associated with traditional biomarkers, including CTL level, TMB, and TGF-β signal, and designed a combined strategy to predict ICB treatment response and prognosis. We suggested that robust prognostic factors should include a comprehensive immune response mechanism and not be associated with immunosuppression. We applied this strategy to TCGA lung cancer samples and obtained an ICB treatment signature (ITS) with 91 genes. In independent ICB treatment cohorts, we demonstrated superior prediction performance of ITS scores over traditional biomarkers.

Drug resistance to targeted drugs including BRAF (32), MAPK (33), and MEK (34) signaling pathways in different cancer types affects patient treatment outcomes. Immunotherapy has been observed to show strong antitumor activity in advanced non-small-cell lung cancer. While the success of immunotherapy was exciting, it was important to note that only a subset of patients will benefit from ICB treatment. Therefore, predicting the treatment response of ICB was a challenge for researchers. At present, various predictive biomarkers have been constructed, but these markers have not achieved satisfactory prediction effects, and the prediction performance of these biomarkers has shown great variability in different datasets (18). In NSCLC, PD-L1 expression was the most commonly used predictive biomarker in routine clinical practice. Some studies have shown that increased PD-L1 expression predicts longer progression-free survival and overall survival after ICB treatment (18). However, PD-L1 expression was not the best biomarker for some patients, because patients with low PD-L1 expression also showed a lasting response (35). Tumor mutation load (TMB) was the second most frequently studied biomarker, where higher TMB was associated with long-lasting clinical benefits and longer overall survival (9–11). Similarly, studies have shown that the tumor immune microenvironment can be used to predict the efficacy of immunotherapy in NSCLC patients. Clinical trials of ICB therapy in NSCLC patients have shown that higher effector T cell level is associated with greater clinical benefit (36). However, none of these markers achieved sufficient predictive power in the ICB dataset we analyzed. The immune response depends on various elements (37), and the complexity of the immune therapy response mechanism led to the fact that prognostic markers designed based on a single mechanism can no longer meet the prediction needs.

Despite the agreement that they are associated with better ICB outcomes, our results suggested that pathways related to cytotoxic T lymphocytes (CTL) differ from pathways related to TMB. Genes related to CTL in LUAD and LUSC represent a broad range of immune response systems including the innate immune system, adaptive immune system, and cytokine pathways. In LUAD and LUSC, TMB-related genes were enriched in cell cycle and RNA metabolism and regulation pathways, respectively. This is consistent with the idea that they represent different immune response mechanisms. CTL plays a killing role in the tumor, and response to ICB treatment requires pre-existing immune microenvironment (38). Tumors with high TMB may be associated with the presence of a large number of neoantigens that can be recognized by CD8+ T cells (39). In addition, we found that the genes related to CTL and TMB contained a large number of genes related to immunosuppressive signature, suggesting that the use of these two types of biomarkers alone would ignore the immune escape mechanism, leading to the instability of prediction performance. We considered that the stable prognostic factors should include a comprehensive response mechanism and exclude the interference of immunosuppressive signals. Based on this idea, we constructed ITS scores in lung cancer to predict ICB treatment response.

ITS is a set of 91 genes identified in LUAD. We did not find a large number of genes associated with both CTL and TMB in LUSC, and one possible explanation is that differences in mutagenicity between the two cancer types lead to different patterns of genomic variation (40). Gene interactions in ITS are involved in cell killing, interferon response, and transcription factor binding. And ITS scores represented comprehensive immune activation characteristics including activated immune cell infiltration, increased mutation load, and TCR diversity. In the independent ICB treatment cohorts, ITS scores were more effective in predicting survival and response to treatment than the single biomarker. Multi-angle evaluation proves that ITS is stable in the application process. This suggests the need to combine multiple immune response mechanisms to predict ICB response.

However, more NSCLC ICB treatment datasets and prospective studies are needed to evaluate the clinical usefulness of ITS. Meanwhile, the ssGSEA method was used to calculate the ITS score in this study. It is not a robust sample classification method to classify samples only according to median ITS value, and more effective methods may be needed to explore the optimal cutoff value. In addition, the complex immune microenvironment of the samples also raises concerns. Gene expression at the bulk level does not take into account intra-tumor heterogeneity, which is an important factor affecting prognosis and treatment. These problems still need further research and exploration in the future.

In summary, this work demonstrated that the combination of biomarkers related to different response mechanisms contributed to better prediction performance of ICB treatment response, and provided a promising immune response biomarker in NSCLC, thus optimizing the treatment regimen of patients.

## Data Availability Statement

Publicly available datasets were analyzed in this study. TCGA data were downloaded from the UCSC Xena platform (https://xenabrowser.net/datapages/) under accession TCGA LUNG. RNA-seq data of NSCLC ICB treatment cohort were downloaded from the European Genome-phenome Archive under accession number EGAS00001003731. RNA-seq data of the Gide et al. study was available from ENA under accession number ERP105482. Other expression data and clinical information used in this study were obtained through the supplementary materials of original publications.

## Author Contributions

Conceptualization: ZJ and JH. Methodology: ZJ, YZ, and JH. Formal computational analysis: ZJ, YZ, and JH. Investigation: ZJ, YZ, and JH. Data visualization: ZJ, YZ, and JH. Original draft: ZJ, YZ, and JH. Writing—review and editing: ZJ, YZ, and JH. All authors contributed to the article and approved the submitted version.

## Conflict of Interest

The authors declare that the research was conducted in the absence of any commercial or financial relationships that could be construed as a potential conflict of interest.

## Publisher’s Note

All claims expressed in this article are solely those of the authors and do not necessarily represent those of their affiliated organizations, or those of the publisher, the editors and the reviewers. Any product that may be evaluated in this article, or claim that may be made by its manufacturer, is not guaranteed or endorsed by the publisher.
