# Supplementary material for: A Combination of Biomarkers Predict Response to Immune Checkpoint Blockade Therapy in Non-Small Cell Lung Cancer
Source: Front Immunol. 2021 Dec 23;12:813331. doi: 10.3389/fimmu.2021.813331 (PMC8733693; doi:10.3389/fimmu.2021.813331)
Supplement: Supplementary file 1 [file DataSheet_1.pdf]

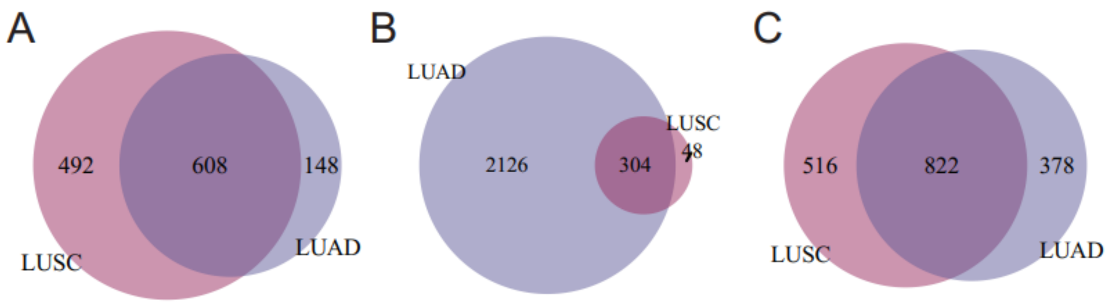

Figure S1. The number of genes associated with (A) CTL, (B) TMB, and (C) TGF-β in LUAD and LUSC.

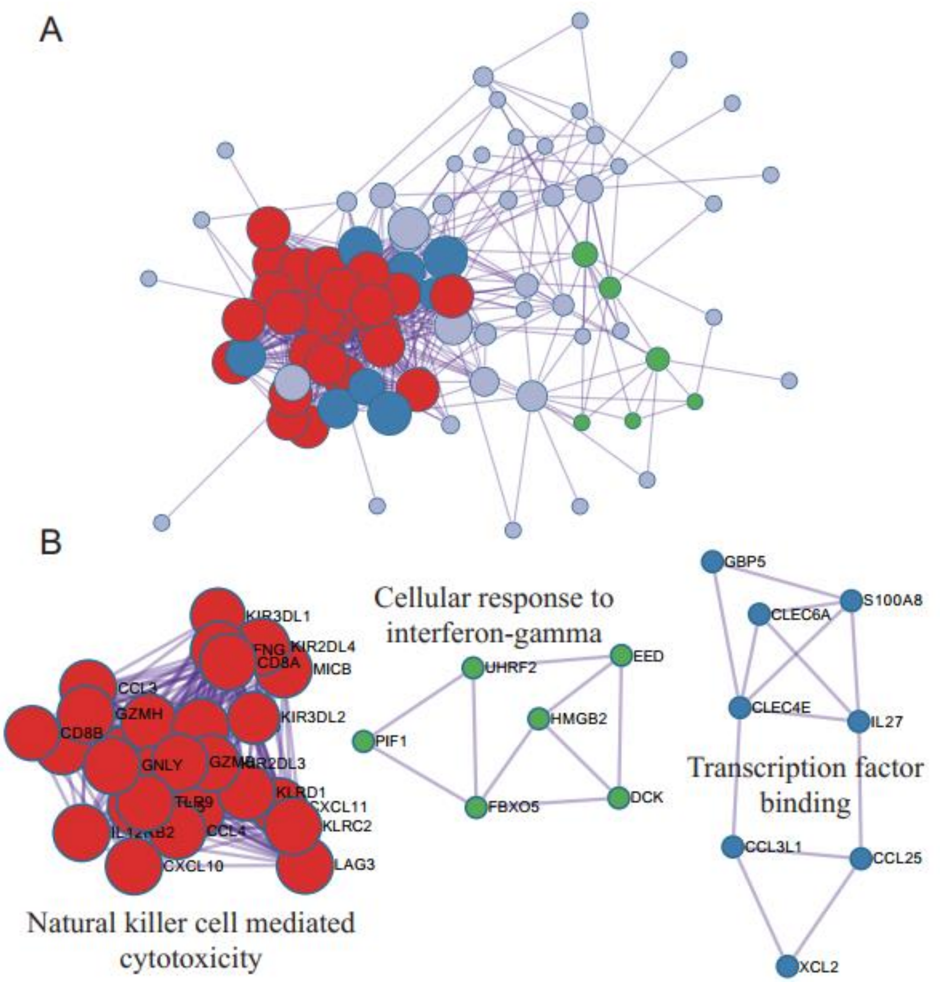

Figure S2. Functions related to ITS. (A) The interaction network of ITS. (B) Function modules related to ITS.

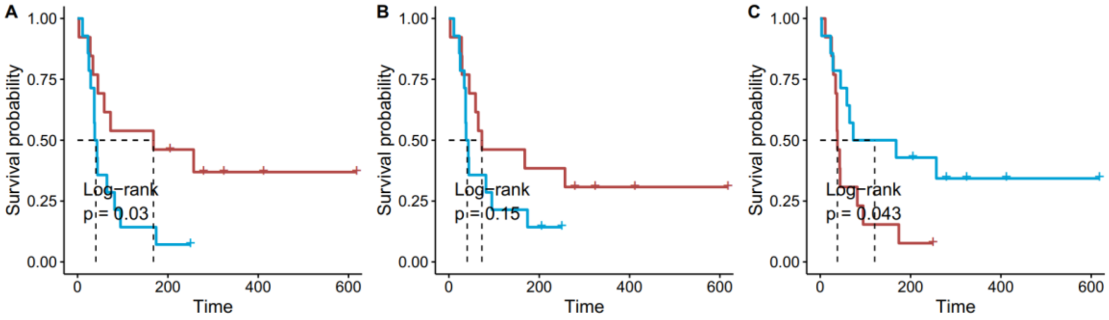

Figure S3. PFS difference between the two groups with high and low ITS scores calculated by (A) GSVA, (B) zscore, and (C) plage methods.

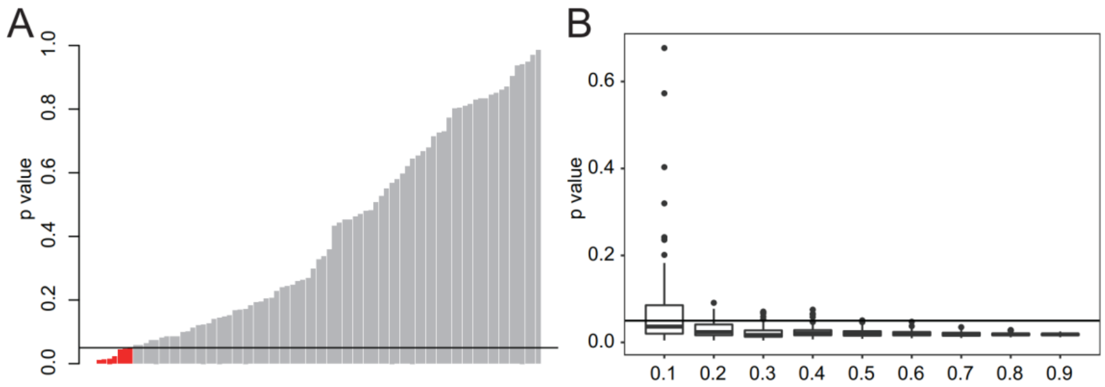

Figure S4. (A) Immunotherapy prognostic ability of each gene in ITS. (B) Immunotherapy prognostic ability of ITS that selected genes randomly in a fixed proportion from 0.1 to 0.9. The sample was repeated 100 times for each proportion.

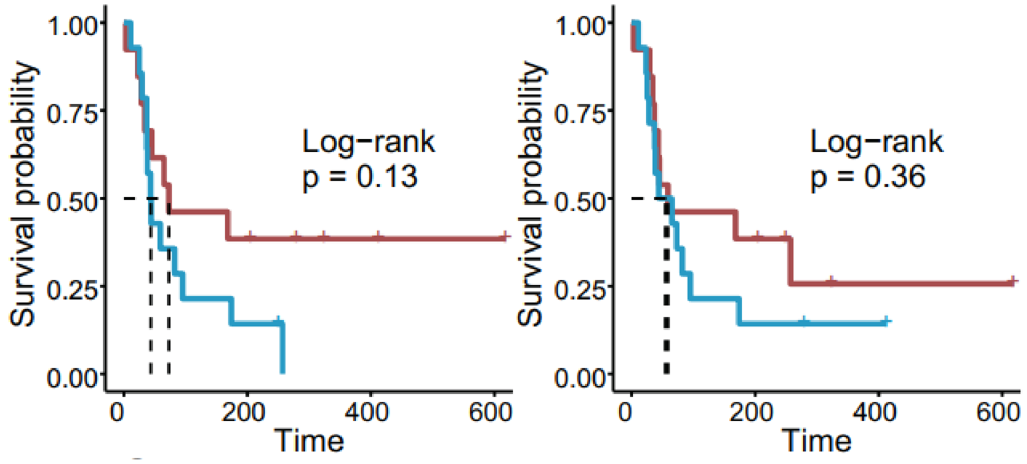

Figure S5. (A) PFS difference between the two groups with high and low CTL levels. (B) PFS difference between the two groups with high and low TGF-β levels.

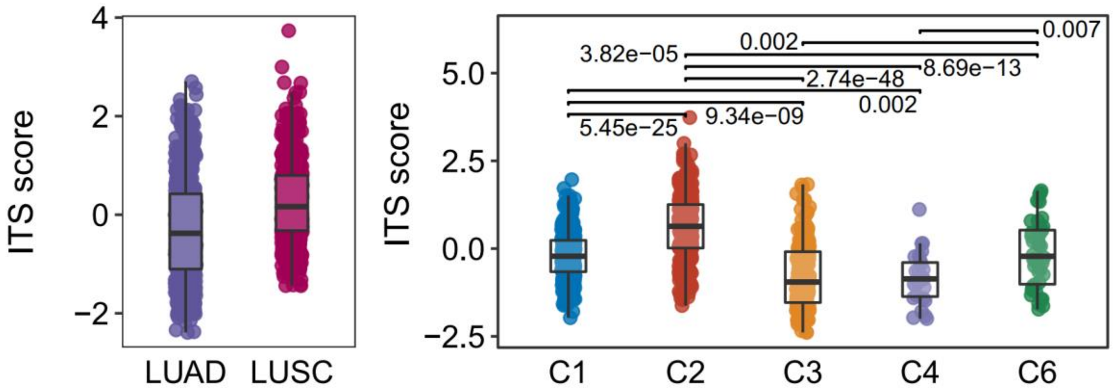

Figure S6. (A) The difference in ITS scores between LUAD and LUSC. (B) The difference in ITS scores between immune subtypes.

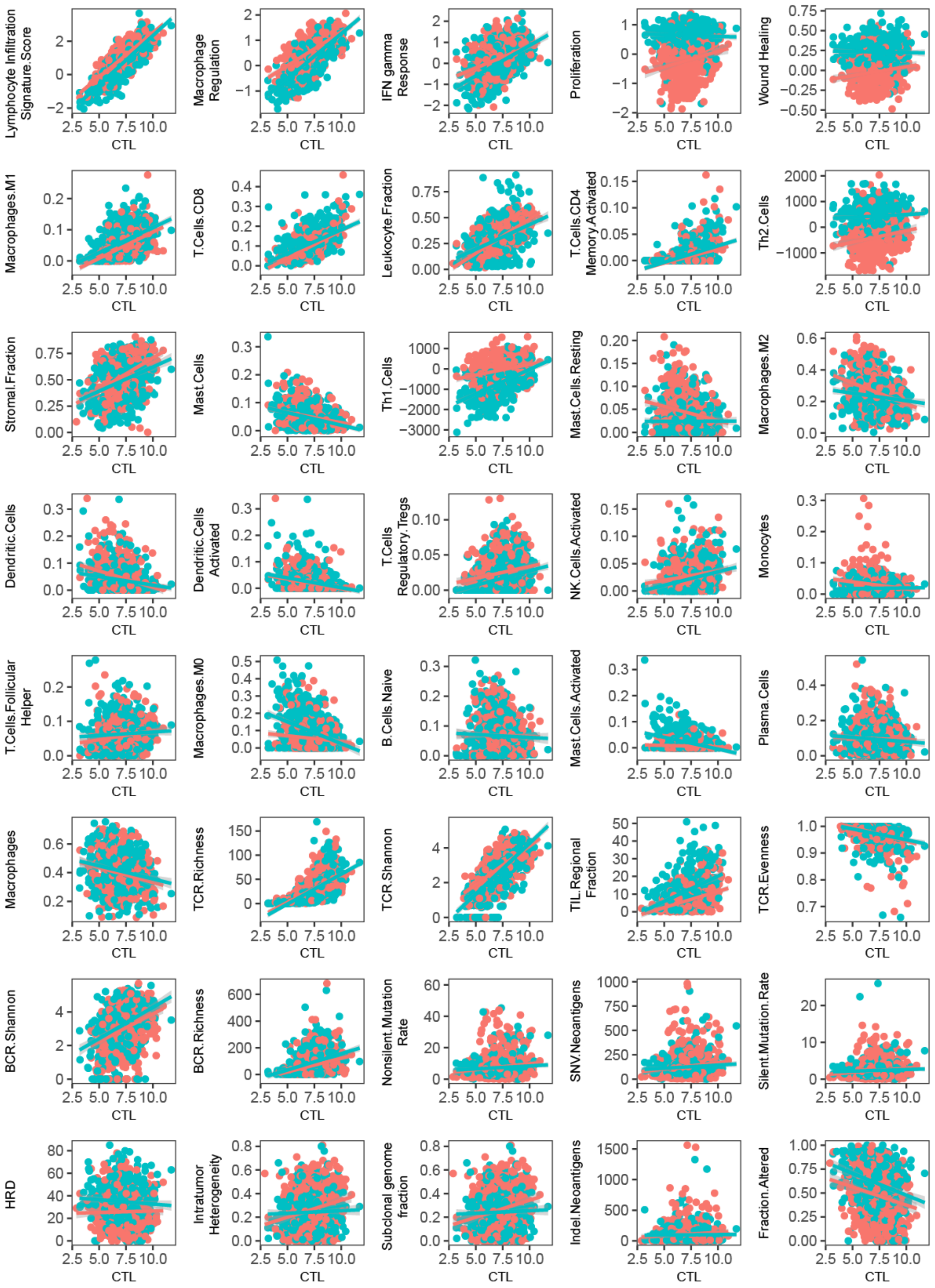

Figure S7. Scatters figures show the association of immune features with ITS. The blue dots represent the LUSC sample and the red dots represent the LUAD sample.
